# Interpreting clinical trial outcomes complicated by placebo response with an assessment of false-negative and true-negative clinical trials in depression using propensity-weighting

**DOI:** 10.1038/s41398-023-02685-y

**Published:** 2023-12-14

**Authors:** Roberto Gomeni, Seth Hopkins, Françoise Bressolle-Gomeni, Maurizio Fava

**Affiliations:** 1 Pharmacometrica, La Fouillade, France; 2 Sumitomo Pharma America. Inc, Marlborough, MA USA; 3 Department of Psychiatry, Massachusetts General Hospital, and Harvard Medical School, Boston, MA USA

**Keywords:** Diseases, Depression

## Abstract

The objective of this study was to evaluate the performances of the propensity score weighted (PSW) methodology in a post-hoc re-analysis of a failed and a negative RCTs in depressive disorders. The conventional study designs, randomizations, and statistical approaches do not account for the baseline distribution of major non-specific prognostic and confounding factors such as the individual propensity to show a placebo effect (PE). Therefore, the conventional analysis approaches implicitly assume that the baseline PE is the same for all subjects in the trial even if this assumption is not supported by our knowledge on the impact of PE on the estimated treatment effect (TE). The consequence of this assumption is an inflation of false negative results (type II error) in presence of a high proportion of subjects with high PE and an inflation of false positive (type I error) in presence of a high proportion of subjects with low PE value. Differently from conventional approaches, the inverse of the PE probability was used as weight in the mixed-effects analysis to assess TE in the PSW analysis. The results of this analysis indicated an enhanced signal of drug response in a failed trial and confirmed the absence of drug effect in a negative trial. This approach can be considered as a reference prospective or post-hoc analysis approach that minimize the risk of inflating either type I or type II error in contrast to what happens in the analyses of RCT studies conducted with the conventional statistical methodology.

## Introduction

The randomized controlled trial (RCT) is considered as the gold standard methodology for assessing efficacy and safety of new treatments in depressive disorders (DD). This approach assumes that randomization prevents systematic and uncontrolled differences across assigned treatment groups and that the response is only driven by the treatment administered.

However, the large number of failed RCTs in DD raises serious concern on the validity of this assumption: the current standard randomization process seems unable to control the higher than expected and uncontrolled level of placebo effect affecting the efficacy assessments. Therefore, the failures of RCTs in DD have become an increasing unresolved issue that affects the clinical development of new antidepressant medications.

As previously pointed out [1], one may classify treated patients in an DD clinical trial based on each participant’s propensity to respond to a given type of treatment. The “D − P − ” population comprises patients who are not responsive to either active treatment (D) and inactive, placebo treatment (P). In DD trials in nonresistant populations, the D − P− group typically represents 30–50% of the populations. The “D + P + ” population comprises patients who are responsive to either active (D) or placebo (P) treatments and represents the intrinsic placebo response rate of the population under investigation. The D + P+ group is typically over 40% in DD trials, making rather small the third population (“D + P-”), which comprises patients who are responsive to active treatment but not to placebo and therefore represents the most informative group of patients. It is therefore not surprising that several meta-analyses indicated that the level of placebo response has a critical prognostic relevance in the assessment of treatment effect (TE defined as the baseline corrected change from placebo in MADRS or HAMD-17 total score) at end of study (EOS) in RCTs conducted in major depressive disorders (MDD) [2–10]. Furthermore, a meta-analysis conducted on 169 antidepressant monotherapy studies and 35 adjunctive polypharmacy studies conducted in MDD, showed that a higher placebo response rate statistically significantly correlates with a low-risk ratio of responding to antidepressant versus placebo [11]. In this framework, TE can be considered as the resultant of a treatment specific and a treatment non-specific response and the individual propensity to respond to any treatment (i.e., the placebo effect usually referred as PE) can be considered as a relevant prognostic factor. The larger is the propensity to respond to non-specific treatment, the lower will be the chance to detect any treatment-specific effect [11, 12].

In this context, new methodological approaches for designing, conducting, and analyzing RCTs are needed for controlling and mitigating the increasing confounding effect of placebo response. The propensity score weighting (PSW) is a novel statistical inference methodology recently proposed for analyzing RCTs in MDD [13, 14]. The aim of PSW is to control for the confounder effect of the intrinsic PE within a given population by achieving balance in PE distribution between exposed and unexposed arms. By accounting for any differences in measured baseline characteristics, the PSW methodology aims to approximate what would have been achieved through a randomization appropriate for insuring a balanced allocation of subjects in the different treatment arms with respect to the PE values at baseline.

The PSW methodology is based on the calculation of propensity, which is the individuals’ probability of showing PE given observations of individual items of the selected clinical scale used for assessing disease severity (i.e., HAMD-17 or MADRS) evaluated between two pre-randomization time points at screening and baseline. The predicted probability was estimated using artificial intelligence (AI) methodologies based on artificial neural network (ANN) approach.

In the present paper, we are presenting a re-analysis of a reportedly failed and a reportedly negative RCTs conducted in DD using the PSW methodology. The individual propensity to PE estimated using an artificial intelligence approach will be used as weight of the individual observations in the mixed-effect model for repeated measures (MMRM) conducted to assess TE.

The objective of the analysis will be to compare the performances of the PSW methodology with the conventional statistical methodology.

## Methods

### Data

The data of two RCTs were re-analyzed used using the propensity weighted approach. The first trial (Study SEP360-029) was a randomized, placebo-controlled, double-dummy, multicenter study of the safety, efficacy, and tolerability of dasotraline, a serotonin-norepinephrine-dopamine reuptake inhibitor, in male and female subjects with DD (ClinicalTrials.gov Identifier: NCT00584974). According to the main criteria for inclusion, male and female subjects between the ages of 18 and 55 years at the time of informed consent who met the DSM-IV criteria for MDD and confirmed by the Mini International Neuropsychiatric Interview (MINI) were included in the trial. Subject meeting criteria for Atypical or Melancholic Features were eligible. The duration of the current episode of MDD was at least 1 month but not longer than 12 months. Subject had at least 1 previous, diagnosed episode of MDD in the past 5 years. MDD was the condition that was chiefly responsible for motivating the subject to seek treatment. The subject had a clinical global impression of severity (CGI-S) score greater than or equal to 4 at screening and baseline. The subject was deemed appropriate by the investigator for medical treatment with venlafaxine for depression.

The study consisted of a screening period, which may have lasted up to 2 weeks; an 8-week (56 days) double-blind treatment period; a 2-week (14 days) wash-out period; and a 1 week (7 days) follow up period. Total subject participation was 13 weeks (91 days). The treatments were 0.5 mg or 2.0 mg dasotraline, 150 mg of venlafaxine, and placebo. Venlafaxine was titrated from 75 mg to 150 mg after 2 weeks. Safety, efficacy, and tolerability were evaluated using clinical observations as well as clinician-rated scales, and subject-administered rating scales. In-clinic visits occurred at Weeks 1, 2, 4, 6, 8, 9, and 11.

A total of 472 subjects (118 per treatment group) were planned to be randomized to complete 400 subjects (100 subjects per treatment group). Subjects were randomized in a 1:1:1:1 ratio to treatment with either 0.5 mg, or 2.0 mg dasotraline, 150 mg venlafaxine, or placebo. This sample size was based on the ability to detect a 3 point improvement in change from baseline in HAM-D-17 for either dasotraline arm compared to placebo, assuming a common standard deviation of 7.5, with 80% power using a 2-sided test at the 0.05 significance level. A total of 514 subjects were randomized to 1 of 4 treatment groups for 11 weeks of treatment including 8 weeks double blind treatment and 3 weeks washout. This study was considered as a negative study as no signal of a clinically meaningful or statistically significant treatment effects for the primary endpoint (i.e., the HAMD-17 total score at week 8) was detected.

The second trial (Study SEP380-201) was a randomized, double-blind, placebo-controlled, parallel-group, fixed-dose study designed to evaluate the efficacy, safety, and tolerability of treatment with non-racemic amisulpride (SEP-4199) monotherapy given as 200 mg/day or 400 mg/day compared with placebo for the treatment of major depressive episode associated with bipolar disorder (ClinicalTrials.gov Identifier: NCT03543410). According to the inclusion criteria, this multi-regional study enrolled outpatients 18–65 years of age who met DSM-5 criteria for bipolar I disorder and were currently experiencing a major depressive episode (≥4 weeks and *<*1 year duration), without psychotic features, but with rapid cycling permitted (*<*8 episodes in the past year). Diagnosis was confirmed by the Structured Clinical Interview for DSM-5, Clinical Trials Version SCID-5-CT. A Montgomery-Åsberg Depression Rating Scale score ≥ 22 and a Young Mania Rating Scale score ≤12 were required at both screening and baseline. Females were enrolled who were unable to become pregnant (postmenopausal or surgically sterile), or who were using a highly effective form of birth control for at least 28 days prior to administration of the first dose of study drug. Patients with type 2 diabetes were eligible for study inclusion if their screening glucose was *<*200 mg/dL, and if their hemoglobin A1c (HbA1c) was ≤7.0%. Patients could be enrolled who were on stable doses (for at least 30 days prior to Baseline) of an oral hypoglycemic, an antihypertensive agent, or thyroid replacement medication. A total of 289 subjects were included in the analysis. The primary efficacy endpoint was the change from placebo in the baseline adjusted Montgomery-Asberg Depression Rating Scale (MADRS) at Week 6 between each non-racemic amisulpride treatment group and the placebo treatment group in the ITT population on subjects who participated in sites located in the US and Europe. A total sample size of 279 evaluable subjects (93 per treatment group: SEP-4199 200 mg/day, SEP-4199 400 mg/day, and placebo) with a 2-sided global alpha of 0.05 was estimated in a power analysis to have about 90% power to reject at least 1 truly significant comparison and about 75% power to reject both truly significant comparisons using the truncated Hochberg (γ = 0.9) procedure, assuming treatment effect sizes of 0.44 for both doses of SEP-4199.

Statistically non-significant improvement in depressive symptoms assessed by the MADRS total score was observed (vs. placebo) for both the 200 mg/day and 400 mg/day dose groups at 6-week study endpoint. In the analysis of the primary endpoint, in patients with bipolar I depression, non-racemic amisulpride showed numerical improvement in the MADRS total score compared to placebo after 6 weeks of treatment. While the study did not meet its primary endpoint, a relatively large improvement in MADRS total score was observed in the placebo group, which may have contributed to the trend level findings of the primary analysis [15]. For this reason, this study was considered as a failed study.

### Placebo response definition

The placebo response was defined as a clinically relevant percent change from baseline (i.e., 50% or more) in the MADRS (study SEP380-201) or HAMD-17 (study SEP360-029) total score in the placebo treated subjects at the study endpoint (EOS = week 6 for SEP380-201 or week 8 for SEP360-029).

### Propensity weighted analysis

The propensity weighted analysis was conducted using a 5-step approach for each study:

1. Selection of the pre-randomization (i.e., screening and baseline) and EOS primary outcome data in subjects randomized to placebo
2. Development of an ANN model using the 10 individual MADRS items (study SEP380-201) or the 17 individual HAMD-17 items (study SEP360-029) change from screening to baseline in subjects assigned to placebo to estimate the probability to be placebo responder at study end.
3. Validation of the ANN model by comparing the model-predicted probability to the observed placebo response.
4. Prediction of the individual probability to have a PE using the pre-randomization data of all subjects randomized in the study (i.e., subjects in the different treatment arms) using the ANN model.
5. Use the inverse individual probability as a weighting factor in the MMRM analysis conducted on the longitudinal clinical scores to estimate the TE.

The original data were randomly split into three datasets for model development and validation:

1. The training set for the ANN model development including 75% of the data in the placebo arm.
2. The validation set for the ANN model including 25% data in the placebo arm not used for model development. The model validation was conducted by comparing the model predictions and data observed in the validation dataset.
3. The working dataset, with the full data set with all subject data in the 3-arms. This dataset was used to provide the individual estimate of the propensity probability applying the validated ANN model to the pre-randomization data of each subject in the 3-arms.

A binary score was associated to each subject: 0 or 1 for absence or presence of placebo response at EOS. The individual MADRS or HAMD-17 items collected at two pre-randomization time points (i.e., at screening and baseline) were used to predict the placebo response at EOS using an ANN methodology [16]. A grid search was conducted for identifying the optimal number of layers and nodes in the ANN model. The optimality criteria were based on the best predictive performance of the model. A bootstrap analysis was applied for estimating the predictive performance and the robustness of the model by computing the area under the receiver operating characteristic (ROC) curve, and the associated 95% confidence interval. The ANN analysis was conducted using the ‘*neuralnet’* library in R [17].

The individual estimate of the propensity probability of PE was finally estimated by applying the ANN model to the pre-randomization data of each subject enrolled in the trials. The inverse of the estimated probability was included as weight in MMRM model used to analyze the longitudinal MADRS or HAMD-17 total scores and to estimate the TE. The MMRM models were implemented in SAS (PROC MIXED, Version 9.4, SAS Institute, Carry, NC, USA), using the change from baseline of the MADRS or HAMD-17 total score. In the MMRM analysis a random effect model was used on the change from baseline value, using an unstructured covariance matrix, time as a classification variable, and baseline measurement as a covariate, baseline x time interaction, and treatment x time interaction. The TE was calculated as the least squares means (LS means) difference at EOS. Based on the MMRM analysis outcomes, between-group effect size at EOS was computed as the absolute value of the LS mean difference from placebo divided by the model estimate of the pooled SD deviation. The reported *p*-values were adjusted for multiplicity using the Tukey methodology.

Two ITT analyses were conducted: the first one (reference) was the conventional analysis (without propensity weight) and the second analysis was the propensity weighted analysis.

## Results

### ANN analysis

**Fig. 1:**
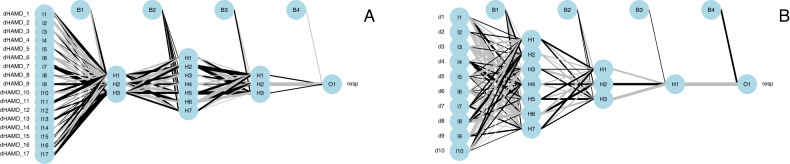
Neural network layout. Final layouts of the ANN models for the analysis conducted using the changes from screening to baseline of the individual items of the MADRS and HAMD-17 clinical scales used as potential predictors of the response (resp = response to placebo) for the SEP360-029 study (**A**) and for the SEP380-201 study (**B**).

The results of the grid search are presented in Table 1 and the final neural network layouts for the ANN analyses are presented in Fig. 1 (panel A for the SEP360-029 study and panel B for SEP380-201 study). In these plots, the first column represents the change from screening to baseline of the individual 10 MADRS or 17 HAMD-17 items considered as predictors of the placebo response (‘resp’), the second column represents the combined items characterizing the first layer, the third column represents the combined items defining the second layer, and the third column represents the item defining the final layer. The lines connecting the nodes are color-coded by sign (black increasing, and gray decreasing effect). The size of the connecting lines in the network is proportional to the relative importance of the information associated to the nodes. A null weight will be associated to variables not relevant for predictions.

**Table 1:** Results of the grid search and of the ANN models for the SEP360-029 study and for the SEP380-201 study.

Study	Nb Layers	Nb Nodes/Layer			ROC	95% CI	
		1	2	3	AUC	Lower	Upper
SEP360-029	3	3	7	3	0.92	0.81	1
SEP380-201	3	7	3	1	0.89	0.73	1

The values of the ROC AUCs for the two studies were statistically greater than the noninformative threshold of 0.5, as indicated by the boundaries of the 95% confidence intervals of the ROC AUC. These results indicated that the two ANN models were able to provide a reliable estimate of the probability to show a non-specific response to a treatment using the individual item score of the MADRS or HAMD-17 scale assessed on two pre-randomization time points.

**Fig. 2:**
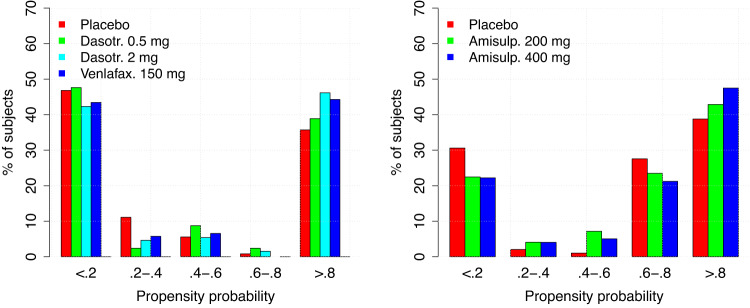
Distribution of the propensity probability to a placebo effect by treatment for the SEP360-029 study (left panel) and for the SEP380-201 study (right panel).

The ANN models were then used to predict the individual propensity to respond to placebo in each subject included in the three arms of the two studies. The percentage of subjects with estimated propensity to respond to non-specific TEs in the intervals <0.2, 0.2–0.4, 0.4–0.6, 0.6–0.8, and >0.8 is presented in Fig. 2.

**Fig. 3:**
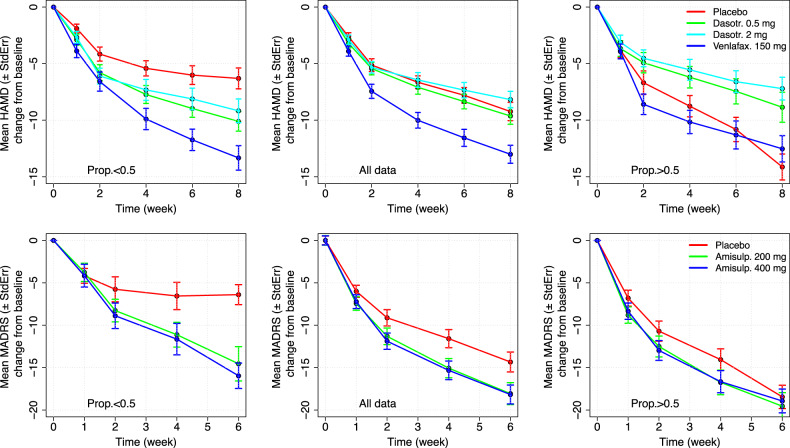
Descriptive statistics (mean ± standard error) on the longitudinal HAMD-17 (study SEP360-029**—**top panel) and MADRS (study SEP380-201 – bottom panel) changes from baseline in the subjects with propensity probability < 0.5 (left panels), all subjects (central panel), and >0.5 (right panels).

The distribution of the propensity probability in the two studies indicates that a large majority of the subjects enrolled in the trials have a high (*p* > 0.8) probability to inflate the response due to a non-specific response to a treatment. Therefore, the size of the TE is expected to be larger when the weighting factor is included in the mixed-effect analysis to account for this unbalance. The descriptive statistics of the HAMD-17 and the MADRS total scores longitudinal changes from baseline in the total population and in the subjects with propensity probability >0.5 and <0.5 is presented in Fig. 3.

The descriptive statistics on the longitudinal changes from baseline of HAMD-17 and MADRS total scores to respond to placebo indicate that the expected detectable signal of a TE is highly reduced in the subjects with high propensity probability to a non-specific response to a treatment (*p* > 0.5).

### MMRM Analysis

**Fig. 4:**
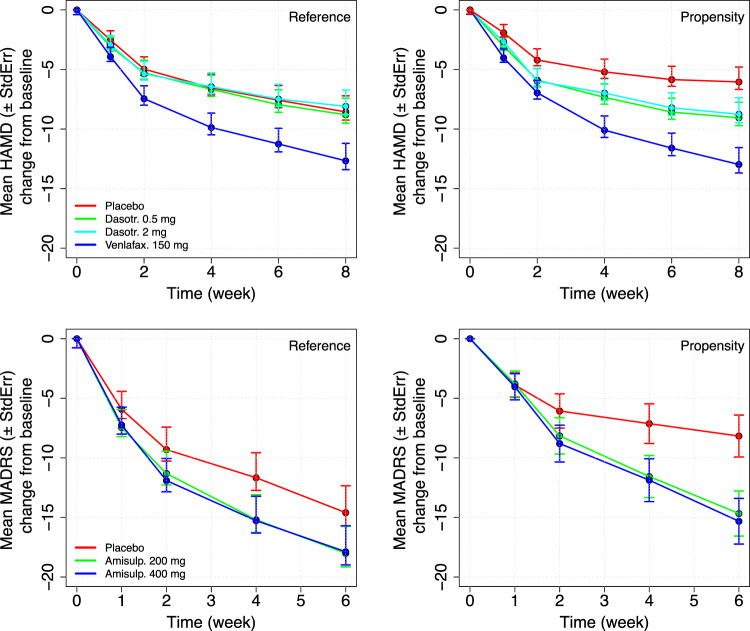
Least Squares Mean (±standard error) of the longitudinal HAMD-17 total score changes from baseline (study SEP360-029 – top panel) and MADRS total score (study SEP380-201**—**bottom panel) estimated using the propensity weighed (left panel) and non-weighted analyses (right panel).

The results of the non-weighted and weighted MMRM analyses with the estimation of the effect sizes are presented in Fig. 4 and in Table 2.

**Table 2:** Treatment effect and effect size in the HAMD-17 and MADRS total score estimated using the propensity weighed and non-weighted analyses.

Study SEP360-029	Comparison	TE	StdErr	P*	SD	Effect_size
**Reference**	Pbo vs. Dasotr. 0.5 mg	0.236	0.985	1.000	7.818	0.030
	Pbo vs. Dasotr. 2.0 mg	−0.466	0.981	1.000	7.849	0.059
	Pbo vs. Venlafax. 150 mg	4.104	1.015	0.0094	7.990	0.514
**Propensity**	Pbo vs. Dasotr. 0.5 mg	3.008	0.916	0.115	7.273	0.414
**Analysis**	Pbo vs. Dasotr. 2.0 mg	2.718	0.953	0.324	7.620	0.357
	Pbo vs. Venlafax. 150 mg	6.928	0.958	<0.0001	7.543	0.918

Study SEP380-201	Comparison	TE	StdErr	p*	SD	Effect_size
**Reference**	Pbo vs. Amisulp. 200 mg	3.411	1.619	0.6188	11.336	0.301
	Pbo vs. Amisulp. 400 mg	3.280	1.592	0.6513	11.170	0.294
**Propensity**	Pbo vs. Amisulp. 200 mg	6.504	1.315	<0.0001	9.208	0.706
**Analysis**	Pbo vs. Amisulp. 400 mg	7.151	1.325	<0.0001	9.295	0.769

TE treatment effect, StdErr standard error, P probability, SD standard deviation.

*P-value adjusted for multiplicity.

The comparison of the TE estimated in the two analyses conducted with the SEP360-029 data indicated the absence of any signal of dasotraline efficacy. Despite the improvement in signal detection, the propensity weighted analysis confirmed the inefficacy of dasotraline at 0.5 mg and 2 mg dose for the treatment of MDD.

Differently from the outcomes of study SEP360-029, the results of the analyses conducted with the study SEP380-201 indicated a strong signal of drug response in the propensity weighted analysis. In this case the TEs and the effect-sizes in the two treatment arms were ~twice larger than the values estimated in the reference (non-weighted) analysis.

## Discussion

The primary objective of this study was to evaluate the performance of the PSW methodology applied to RCTs presenting negative of border-line results and to verify that the PSW methodology was not inflating the type I or type II error.

In the PSW approach all subjects randomized in the trial were included in the analysis consistently with the intention-to-treat (ITT) paradigm. Therefore, PSW cannot be considered as an enrichment strategy as no data of any subject were excluded from the analysis in the attempt remove subjects who have large PE.

The large number of meta-analyses conducted on many RCTs in MDD strongly support the assumption that the estimated TE value is highly correlated with the distribution of the treatment not-specific response (PE): the higher the PE, the lower will be the estimated TE value [11].

The conventional statistical model based on a liner mixed-effect longitudinal analysis approach (without any individual weight), does not account for the baseline distribution of PE as a prognostic factor. Therefore, the statistical model implicitly assumes that the baseline PE is the same for all subjects enrolled in the trial even if this assumption is not supported by our knowledge on the impact of PE on the estimated TE. There is a clear evidence of variability in the proportion of P + D+ subjects within an MDD trial and this may clearly confound the results. In the case of study SEP380-201, for example, the MADRS change of almost 15 points reflects an extraordinarily high rate of placebo response. Therefore, the consequence of the conventional statistical model’s assumption is an inflation of false negative results (type II error) in presence of a high proportion of subjects with high PE, as in the case of study SEP380-201. Conversely, the conventional statistical model can lead to an inflation of false positive results (type I error) in presence of a higher proportion of subjects with low PE value.

The PSW methodology currently used in epidemiological and social science studies has been proposed as a novel approach to better control the potential negative effect of unbalanced distribution of PE in the assessment of the TE and the effect size in RCTs. Recently, it was adopted in a regulatory setting by the FDA, where it was used in observational studies to support marketing applications for medical devices [18–21].

An important question concerning the potential inflation of the type II error (the risk of false negative results) associated with the outcomes of the PSW methodology must be addressed. When applied to randomized controlled trials, PSW ensures balance between groups at the time of randomization, accounts for chance imbalances in observed randomization, and generalizes target results to target populations [22]. Therefore, the use of PSW can be considered as a reference approach that minimizes the risk of inflating either type I or type II error at variance to what happens in the analyses of RCT studies conducted with the conventional statistical methodology.

There is great flexibility in how the propensity scores methodology can be implemented. For example, different criteria can be used for identifying placebo responders in the ANN modeling, and each different criterion can lead to a different estimate of individual probability of PE. This flexibility can lead to a multiple testing approach in the attempt to identify the analysis option providing the smallest *p*-value and such a strategy must inevitably inflate type I error rates. Hence, the prospective definition of any statistical modeling details of the statistical treatment of propensity scores has to be prospectively defined in the statistical analysis plan in order to avoid risk of very serious over-inflation of type I error rates [23, 24].

The re-analysis of the data using the PSW methodology increased substantially the separation of active drug from placebo in study SEP380-201, indicating that the results initially found using an unadjusted analysis were mainly driven by the excessively high percent of subjects with high PE values (i.e., >50% as reported in Fig. 2).

The estimated TE and effect size derived using the current statistical methodologies represent only a working estimate of these values. This estimate is strongly correlated with the level of imbalance in the individual propensity distribution consistently with the expected effect of low/high placebo response on TE [11].

The re-analysis of the data of study SEP360-029 confirmed the results initially found using an unadjusted analysis despite the high percent of subjects with high PE values comparable to the % of subjects in the study SEP380-201. Despite the adjustment of potential unbalance in PE and consistently with the descriptive analysis on the longitudinal HAMD-17 total score in study SEP360-029 did not reveal any separation between placebo and any dose of dasotraline (0.5 mg and 2 mg), while confirming the efficacy of the active comparator (venlafaxine). These findings indicate that the PSW methodology did not artefactually detect a treatment effect signal when this signal was not present.

In the present analyses, the change in the individual MADRS or HAMD scores from screening to baseline have been used as potential predictors of placebo response. Many additional or alternative potential pre-randomization parameters can be also considered such as the demographic data, the habits and quality of life, or the disease-related information, etc. in the attempt to improve the overall predictive performance of the ANN model. For simplicity, we decided to limit our exploration to the individual items of the HAMD and MADRS scale as these items are assumed to capture specific and independent symptoms of depression, and, more important, the total score of these clinical scales is used to estimate the clinical response at study end.

The PSW methodology can be prospectively applied to any RCT designed and conducted using conventional methodologies when: (i) the RCT was designed to collect screening and baseline data, (ii) the criteria for assessing the clinical response to placebo were pre-specified in the analysis plan, (iii) the criteria for implementing and qualifying the predictive performance of the ANN model were defined in the analysis plan.

As discussed in [14], the individual propensity weighted scores estimated in one RCT cannot be generalized and prospectively applied to the data of other RCTs even if the other RCTs have similar designs. This because, the individual propensity to respond to placebo is associated with the individual expectations. This varies from individual to individual as it is associated with study specific implementation and conduction factors. For this reason the ANN model for the PSW estimation has to be conducted, qualified and validated with the data of each trial.

In conclusion, the PSW methodology aims to hit a so far elusive sweet spot by decreasing type II error (fewer false negative studies) while not enhancing false positive studies (type I error). Additional re-analyses of different studies are needed to understand the potential of the PSW methodology better, considering also the risk of publication bias in post-hoc analysis. However, this work provides a new analysis tool towards mitigating the known challenges posed by MDD clinical trials nicely benchmarked by Freeman and colleagues [25].

## Data Availability

The corresponding author will make the files used in the current study available upon receipt of an appropriate request.
